# First person – Kaisa Pakari

**DOI:** 10.1242/bio.062320

**Published:** 2025-11-11

## Abstract

First Person is a series of interviews with the first authors of a selection of papers published in Biology Open, helping researchers promote themselves alongside their papers. Kaisa Pakari is first author on ‘ Establishing an auxin-inducible GFP nanobody-based acute protein knockdown system to mimic hypomorphic mutations during early medaka embryogenesis’, published in BiO. Kaisa is a postdoc in the lab of Thomas Thumberger at the Centre for Organismal Studies (COS), Heidelberg, Germany, investigating glycosylation disorders using medaka fish as a model system.


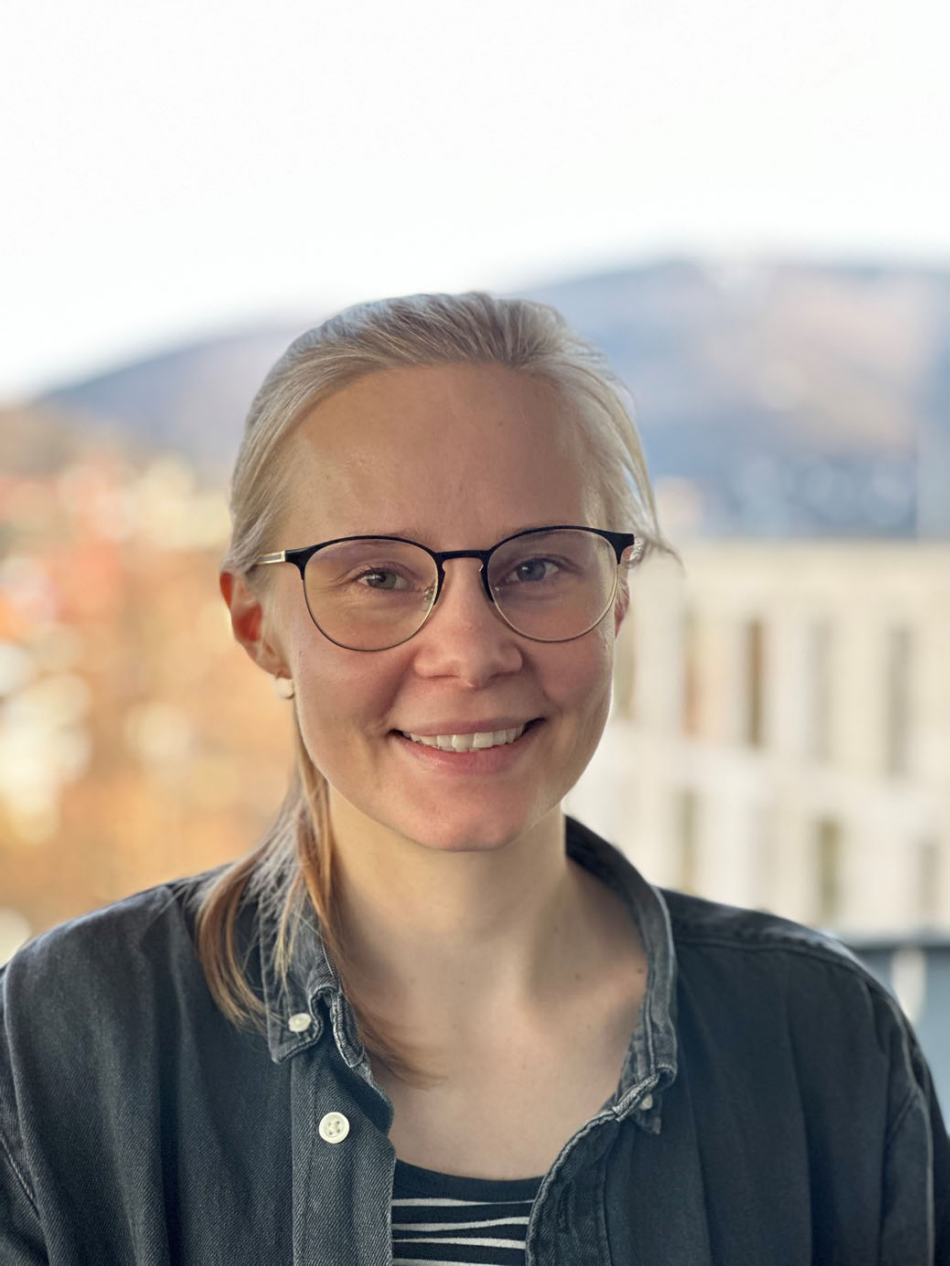


**Kaisa Pakari**

**Describe your scientific journey and your current research focus**

My scientific journey began with a bachelor's degree in molecular biosciences and continued with a master's in molecular biotechnology. I have always been excited by various fields of biology and used my studies as an opportunity to explore different topics and techniques. During several lab rotations across different research fields, I discovered my interest in developing and applying genetic tools to study complex biological processes in model organisms as well as human diseases. Currently, my research focuses on modelling rare human metabolic diseases called congenital disorders of glycosylation (CDG), to better understand disease mechanisms, as there is currently no available cure. I am motivated by contributing to our understanding of how biological processes work and what happens when they go wrong, with the ultimate goal of improving human health. Being part of this process is very rewarding and inspires me to work in science.

**Who or what inspired you to become a scientist?**

There wasn't one specific person or moment that inspired me to become a scientist. After finishing school, I considered careers as an air traffic controller or a media designer. However, I have always been fascinated by genetics and working in a lab has been a childhood dream of mine, which is why I decided to study biosciences. Over time, I realised how much I enjoyed the creative side of research. In many ways, science combines both aspects I always liked: problem-solving and creativity. As a scientist, you get to design experiments, prepare presentations and communicate your work visually. What I appreciate most is the collaboration and exchange of ideas with colleagues and mentors, as well as the independence that comes with being a scientist. Looking back, I'm happy that I followed this path because it turned out to be the perfect balance of analytical and creative work for me.

**How would you explain the main finding of your paper?**

Some enzymes are so essential for cell function that their complete loss would be incompatible with life. Therefore, studying their role and function requires tools that reduce enzyme activity, either by genetic modifications or by lowering the amount of protein. In this study, we established a so-called ‘degron’ system for use in the medaka fish (*Oryzias latipes*), which allows us to control how much of a specific protein is present. The system makes it possible to remove certain proteins in a controlled, inducible manner. Using this method, we reduced the amount of an enzyme called Pmm2, mimicking the reduced activity of PMM2, which is reported in patients suffering from a rare human disease called congenital disorder of glycosylation (PMM2-CDG). Modelling this disease in an animal system like the medaka fish allows us to better understand how the disease develops and to explore potential therapeutic approaches.

**Figure:**
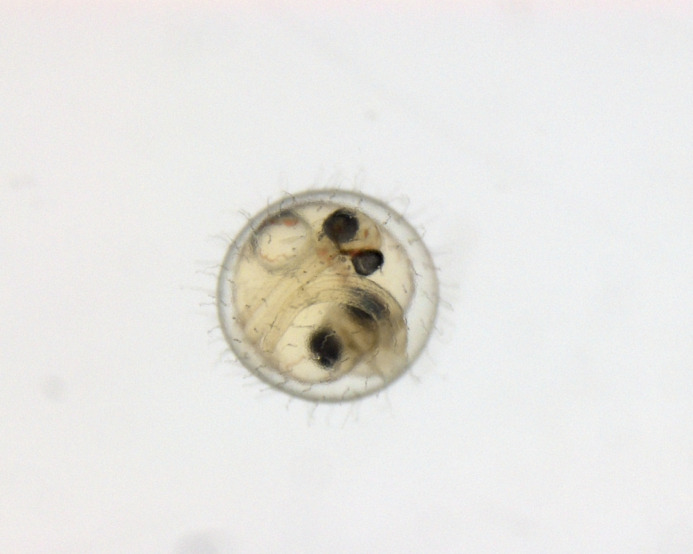
**Conjoined medaka twins – a rare find, like a four-leaf clover in the lab**.

**What are the potential implications of this finding for your field of research?**

Early embryogenesis is difficult to study in humans and mammals in general, but in medaka these stages are accessible due to the extra-uterine development and transparency of the fish embryos. This is particularly valuable for investigating the early onset of diseases. The establishment of this degron system in medaka has significant implications for studying glycosylation disorders (here PMM2-CDG). As a tool, the degron system can be applied to any available GFP-tagged medaka lines to study other disease mechanisms in the future.

**Which part of this research project was the most rewarding?**

The most rewarding part of this project was sharing the method at conferences through talks and poster sessions. It was really exciting to see how such a tool sparks interest among other researchers. Receiving positive feedback made me appreciate the broader impact and potential of this approach.

**What piece of advice would you give to the next generation of researchers?**

Science can be tough sometimes, there will be long days, failed experiments and moments that are very challenging. My advice to the next generation of researchers is to stay connected with your peers because sharing experiences and supporting each other makes a huge difference. Going to conferences and talking about your science can also be incredibly motivating. It reminds you that your work matters and that you are part of a larger community.

**What's next for you?**

Right now, I am focusing on publishing the main findings of my PhD and wrapping up remaining projects. Working as a bridging postdoc also gives me time to prepare for my next steps and shape my future directions.
